# Hydrogen Sulfide Suppresses Skin Fibroblast Proliferation *via* Oxidative Stress Alleviation and Necroptosis Inhibition

**DOI:** 10.1155/2022/7434733

**Published:** 2022-06-21

**Authors:** Ling Li, Ziying He, Yue Zhu, Qiyan Shen, Shengju Yang, Shuanglin Cao

**Affiliations:** ^1^Department of Dermatology, Affiliated Hospital of Nantong University, Nantong 226001, China; ^2^Yancheng No.1 People's Hospital, Yancheng 224001, China

## Abstract

Keloid is a common dermatofibrotic disease with excessive skin fibroblast proliferation. Hydrogen sulfide (H_2_S) as the third gasotransmitter improves fibrosis of various organs and tissues. Our study is aimed at investigating the effects and possible mechanisms of H_2_S on skin fibroblast proliferation. Scar tissues from six patients with keloid and discarded skin tissue from six normal control patients were collected after surgery, respectively. Plasma H_2_S content and skin H_2_S production in patients with keloid were measured. Keloid fibroblasts and transforming growth factor-*β*_1_- (TGF-*β*_1_, 10 ng/mL) stimulated normal skin fibroblasts were pretreated with H_2_S donor as NaHS (50 *μ*M) for 4 h. Cell migration after scratch was assessed. The expressions of *α*-smooth muscle actin (*α*-SMA), proliferating cell nuclear antigen (PCNA), collagen I, and collagen III were detected by immunofluorescence, real-time PCR, and/or Western blot. Intracellular superoxide anion and mitochondrial superoxide were evaluated by dihydroethidium (DHE) and MitoSOX staining, respectively. Mitochondrial membrane potential was detected by JC-1 staining. Apoptotic cells were detected by TDT-mediated dUTP nick end labeling (TUNEL). The expressions of receptor interacting protein kinase 1 (RIPK1), RIPK3, and mixed lineage kinase domain-like protein (MLKL) were measured by Western blot. We found that H_2_S production was impaired in both the plasma and skin of patients with keloid. In keloid fibroblasts and TGF-*β*_1_-stimulated normal skin fibroblasts, exogenous H_2_S supplementation suppressed the expressions of *α*-SMA, PCNA, collagen I, and collagen III, reduced intracellular superoxide anion and mitochondrial superoxide, improved the mitochondrial membrane potential, decreased the positive rate of TUNEL staining, and inhibited RIPK1 and RIPK3 expression as well as MLKL phosphorylation. Overall, H_2_S suppressed skin fibroblast proliferation *via* oxidative stress alleviation and necroptosis inhibition.

## 1. Introduction

Keloid is a common dermatofibrotic disease with main characteristics of excessive skin fibroblast proliferation and extracellular matrix accumulation [1]. Keloid is usually regarded as a benign skin tumor according to clinical manifestation, gene phenotype, pathological features, and prognosis [2]. Keloid has become a cosmetic problem because most protruding scar tissues are beyond the epidermis with obvious pigmentation. In addition, patients suffering from keloid usually feel pruritus and pain, which even cause psychological problems such as inferiority and anxiety [3]. Keloid can be treated by corticosteroid injection, radiotherapy, cryotherapy, 5-fluorouracil, tamponade, laser, and surgery [4]. However, there is still a high recurrence rate because of the lack of “gold standard” for keloid treatment [5]. Therefore, seeking effective interventions for keloid is a hot topic in clinic.

Transforming growth factor-*β*_1_ (TGF-*β*_1_) is recognized as one of the most important factors for fibrosis [6]. Previous studies have confirmed that the content of TGF-*β*_1_ in keloid was significantly increased, while TGF-*β*_1_ inhibition prevented keloid fibroblast proliferation and attenuated extracellular matrix synthesis [7]. However, TGF-*β*_1_ blockage is prone to causing extensive damage by diversified biological effects due to nonspecific inhibition. It is necessary to explore novel strategies to suppress keloid fibroblast proliferation and improve dermatofibrotic diseases.

Hydrogen sulfide (H_2_S) is the third gasotransmitter with multiple functions, which plays a vital role in a variety of physiological and pathological processes [8]. H_2_S is mainly generated by catalysis of cystathionine *γ*-lyase (CSE) in skin [9, 10]. Previous researches showed that H_2_S promoted skin wound healing by inflammation inhibition, oxidative stress suppressing, and vascular endothelial growth factor (VEGF) enhancement [11–14]. H_2_S supplement significantly alleviated skin pruritus in mice [15]. H_2_S also induced apoptosis and blocked the activation of nuclear factors-*κ*B (NF-*κ*B) to inhibit melanoma cells growth and prevent tumor progression [16, 17]. That is to say, H_2_S showed protective effects against various skin diseases. Additionally, previous studies found that H_2_S inhibited the activation of fibrosis-related cells and cytokine expression, then alleviated renal fibrosis, and improved renal function in the rats with renal sclerosis caused by renal tubular epithelial mesenchymal transition (EMT) [18]. Exogenous H_2_S significantly decreased the level of TGF-*β*_1_*in vivo*, increased the expression of aortic elastin, and prevented diabetic nephropathy arteriosclerosis in rats [19]. H_2_S improved myocardial fibrosis *via* suppressing the TGF-*β*_1_/Smad signaling pathway [20]. H_2_S inhibited the TGF-*β*_1_/Smad signaling pathway, improved lung function, and alleviated dermatitis, pulmonary alveoli injury, and chronic obstructive pulmonary fibrosis in cigarette smoke-induced chronic obstructive pulmonary disease of rats [21, 22]. The evidence demonstrated that H_2_S had potential effects on fibrotic diseases [23]. However, the possible effect of H_2_S on keloid and other dermatofibrotic disease remains unknown. A recent study showed that the impairment of endogenous H_2_S aggravated mitochondrial damage, increased reactive oxygen species (ROS), and induced necroptosis to aggravate diabetic cardiomyopathy. Exogenous H_2_S improved mitochondrial function, inhibited oxidative stress, and reduced necroptosis to improve diabetic cardiomyopathy [24]. Moreover, H_2_S donors inhibited necroptosis and alleviated hypoxia-induced myocardial fibroblast proliferation depending on sirtuin 3 [25]. However, whether oxidative stress and necroptosis were involved in the possible effect of H_2_S on skin fibroblast proliferation has not been known well.

Therefore, NaHS, a common H_2_S donor, was applied to investigate whether exogenous H_2_S supplementation could reverse excessive proliferation in keloid fibroblasts and in TGF-*β*_1_-induced normal skin fibroblasts. In addition, the mechanisms of H_2_S on skin fibroblast proliferation were further explored from the perspective of oxidative stress and necroptosis. It is beneficial to provide a novel strategy for the prevention and treatment of dermatofibrotic diseases including keloid, which has a potential clinical transformation prospect.

## 2. Materials and Methods

### 2.1. Collection of Human Skin Samples

Scar tissues from six patients with keloid and discarded skin tissue from six normal control patients without any skin diseases were collected after surgery, respectively. All cases of keloid and normal control were free of infection, tumor, cardiovascular diseases, renal diseases, diabetes mellitus, and other systemic diseases. All patients were not subjected to any local drug injection, radiation, or surgical treatment within the latest 2 years, and any topical drug treatment within the latest 1 month. Plasma was also collected from the above patients. The study strictly followed the requirements of the Declaration of Helsinki. All patients signed a written informed consent. The research was approved by the ethics committee of Affiliated Hospital of Nantong University (No. 2020-L146).

### 2.2. Determination of H_2_S Levels in Plasma and Skin

After polarization, H_2_S-specific microelectrode was inserted into the plasma. The current change was measured by the connected free radical analyzer (World Precision Instruments Inc., Sarasota, Florida, USA). H_2_S concentration was calculated according to the standard curve drawn by the standard concentration and corresponding currents. The concentration of H_2_S in plasma was calibrated with that in normal control.

The collected skin samples were cut into pieces and homogenized (1/10, *w*/*v*) in K_2_PO_4_ (100 mM). Then 460 *μ*L homogenization buffer was taken, and 20 *μ*L L-cysteine (10 mM) and 20 *μ*L pyridoxal-5′-phosphate (2 mM) were sequentially added. The H_2_S level was measured as the above methods. H_2_S production in the skin was calculated according to the standard curve and normalized with protein concentration, which were calibrated with that in normal control.

### 2.3. Primary Skin Fibroblasts Culture and Treatment

After removing epidermis and surrounding fat, skin samples were cut into pieces of 1 mm^3^, cultured in Dulbecco's modified Eagle's medium (DMEM, Gibco, Grand Island, New York, USA) with 10% fetal bovine serum (FBS, Gibco, Grand Island, New York, USA) and subcultured for further experiments. Keloid fibroblasts from keloid were pretreated with NaHS (50 *μ*M, Sigma-Aldrich, St. Louis, MO) for 4 h. Skin fibroblasts from normal skin were pretreated with NaHS (50 *μ*M) for 4 h followed with or without TGF-*β*_1_ (10 ng/ml, PeproTech, Suzhou, China) stimulation for 12 h. The same amount of culture medium was given as a control group.

### 2.4. Cell Migration Assay after Scratch

After the above treatment, skin fibroblasts were scratched with pipette tips of 10 *μ*L, and the shedding cells were washed out with PBS. Cell migration was recorded by photograph after scratch for 0, 4, 8, 12, and 24 h. The migration rate of cells was calculated as the ratio of the migrated area to the original area.

### 2.5. Immunofluorescent Staining

After incubation with *α*-smooth muscle actin (*α*-SMA, 1 : 100; Bosterbio, Wuhan, China), proliferating cell nuclear antigen (PCNA, 1 : 100; Abclonal, Wuhan, China), and collagen I and collagen III (1 : 100; Bosterbio, Wuhan, China) antibodies overnight at 4°C, skin fibroblasts were incubated with Cy3- or Alexa Fluor 488-conjugated IgG (1 : 500; Beyotime, Shanghai, China) for 2 h in the dark. Nuclei were stained by 4′,6-diamidino-2-phenylindole (DAPI, Beyotime, Shanghai, China). The fluorescence was observed and photographed with a laser confocal microscope (Leica, Wetzlar, Germany). The fluorescence intensity was evaluated with the ImageJ software and expressed as the fold of that in control group.

### 2.6. Dihydroethidium (DHE) Staining

After incubation with DHE (2 *μ*M, Beyotime, Shanghai, China) at 37°C for 30 min in the dark, the superoxide anion production in skin fibroblasts was evaluated as DHE fluorescence intensity which were observed and photographed with a laser confocal microscope.

### 2.7. Mitochondrial Superoxide Detection

Mitochondrial superoxide in skin fibroblasts was detected with MitoSOX (5 *μ*M, YEASEN, Shanghai, China) staining. MitoTracker Green (200 nM, Beyotime, Shanghai, China) was incubated for mitochondria colocation at 37°C for 20 min in the dark. The fluorescence was observed and photographed with a laser confocal microscope.

### 2.8. Mitochondrial Membrane Potential (Δ*ψ*m) Detection

After incubation with JC-1 staining solution (Beyotime, Shanghai, China) at 37°C for 20 min in the dark, the fluorescence in skin fibroblasts was observed with a laser confocal microscope at 495/519 nm wavelengths for JC-1 Monomers and 550/570 nm wavelengths for JC-1 Aggregates, respectively.

### 2.9. TdT-Mediated dUTP Nick End Labeling (TUNEL) Staining

After incubation with PBS containing 0.5% Triton X-100 at room temperature for 5 min, the skin fibroblasts were incubated with the TUNEL detection kit (Beyotime, Shanghai, China) containing terminal deoxynucleotidyl transferase (TdT) and fluorescence agent at 37°C for 60 min in the dark. The fluorescence was photographed with a laser confocal microscope. The TUNEL-positive cells were calculated as the fold of the control group.

### 2.10. Real-Time PCR

Total RNA was extracted from skin fibroblasts with TRIzol. Then, RNA samples were subjected to reverse transcription with the following procedure: 37°C for 15 min, 85°C for 5 sec, and 4°C forever. The cDNA was mixed with SYBR Green qPCR mixture (Takara, Otsu, Shiga, Japan) for further amplification (ABI 7500, ABI, Carlsbad, CA, USA). The primer sequences were listed as follows: *α*-SMA, sense 5′-GCGATCTCACCGACTACCTG-3′ and antisense 5′-GCCGACTCCATACCGATGAA-3′; collagen I, sense 5′-AGACATCCCACCAATCACCT-3′ and antisense 5′-CGTCATCGCACAACACCTT-3′; collagen III, sense 5′-TGGCATCAAAGGACATCG-3′ and antisense 5′-CATAATACGGGGCAAAACC-3′; and 18S, sense 5′-AGTCCCTGCCCTTTGTACACA-3′ and antisense 5′-CGATCCGAGGGCCTCACTA-3′. The relative mRNA expression levels were calculated by the *ΔΔ*Ct method.

### 2.11. Western Blot

The proteins from skin fibroblasts were separated by the sodium dodecyl sulphate- (SDS-) polyacrylamide gel electrophoresis (PAGE) and transferred to a polyvinylidene fluoride (PVDF, Millipore, Billerica, MA, USA) membrane. The membranes were blocked by 5% milk without fat for 2 h, then incubated with anti-*α*-SMA (1 : 2000; Bosterbio, Wuhan, China), anti-PCNA (1 : 2000; ABclonal, Wuhan, China), anti-receptor interacting protein kinase 1 (RIPK1), RIPK3, anti-mixed lineage kinase domain like protein (MLKL), p-MLKL (1 : 1000; Cell Signaling Technology, Danvers, MA, USA), anti-GAPDH (1 : 3000; Sigma-Aldrich, St. Louis, MO, USA), anti-*β*-tubulin (1 : 3000; CMCTAG, Milwaukee, WI, USA), or anti-*β*-actin (1 : 5000; ABclonal, Wuhan, China) antibodies at 4°C overnight. Next, the secondary antibody (1 : 5000; Beyotime, Shanghai, china) was incubated for 2 h at room temperature. The protein bands were visualized on the membrane with enhanced chemiluminescence (ECL, Thermo Fisher Scientific Inc., Rockford, IL, USA) solution.

### 2.12. Statistical Analysis

All data were expressed as the mean ± standard error of the mean (SEM) and analyzed by *t* test or one-way ANOVA followed by the Student-Newman-Keuls (SNK) test with the Stata 13.0 software. The value of *P* less than 0.05 was considered significant difference.

## 3. Results

### 3.1. H_2_S Production Is Impaired in Patients with Keloid

**Figure 1:**
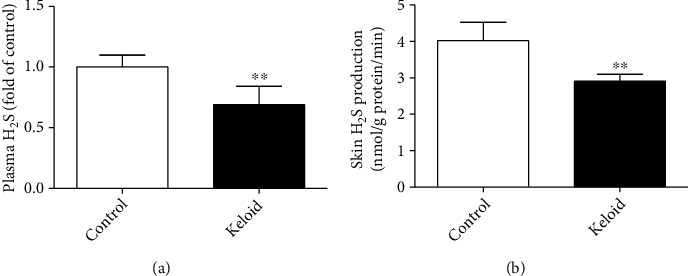
H_2_S production was impaired in patients with keloid. Scar tissues from patients with keloid (Keloid) and discarded skin tissue from normal control patients (Control) were collected after surgery, respectively. Plasma was also collected from above patients. (a) Plasma H_2_S content was detected. (b) Skin H_2_S production was assessed. ^∗∗^*P* < 0.01 vs. control, *n* = 6.

H_2_S levels were measured in plasma and skin tissues of patients. There were lower plasma H_2_S content and skin H_2_S production in patients with keloid than that in normal control (*P* < 0.01) (Figure 1), suggesting that H_2_S production was impaired in patients with keloid.

### 3.2. NaHS Inhibits Keloid Fibroblast Migration after Scratch

**Figure 2:**
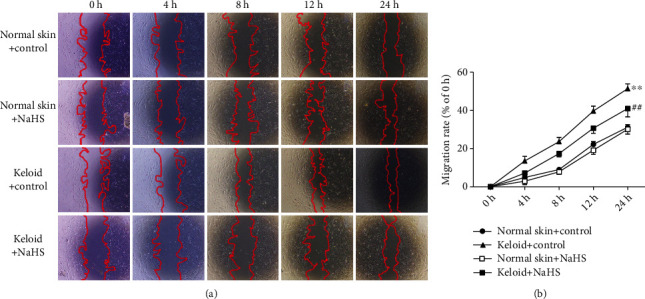
NaHS inhibited keloid fibroblast migration after scratch. Skin fibroblasts were extracted from skin tissues of normal controls (normal skin) and scar tissue from patients with keloid (keloid), respectively. After pretreatment with NaHS (50 *μ*M) or the same amount of culture medium (control) for 4 h, the fresh culture medium was replaced. The cells were scratched with a 10 *μ*L pipette tip 12 h later. (a) Photos were taken after scratch for 0 h, 4 h, 8 h, 12 h, and 24 h. (b) The percentage of cell migration area to initial scratch area at different time. ^∗∗^*P* < 0.01 vs. normal skin + control; ^##^*P* < 0.01 vs. keloid + control, *n* = 6.

However, whether impaired H_2_S production was critical in the pathological process of keloid was unknown. Next, we investigated the influence of restoring H_2_S level on keloid fibroblast proliferation. The scratch migration assay showed that the migration rate of keloid fibroblasts after scratch was faster than that of normal skin fibroblasts (*P* < 0.01), which was significantly reduced by NaHS pretreatment (*P* < 0.01) (Figure 2). It suggested that exogenous H_2_S supplementation inhibited keloid fibroblast migration after scratch.

### 3.3. NaHS Suppresses *α*-SMA Expression in Keloid Fibroblasts

**Figure 3:**
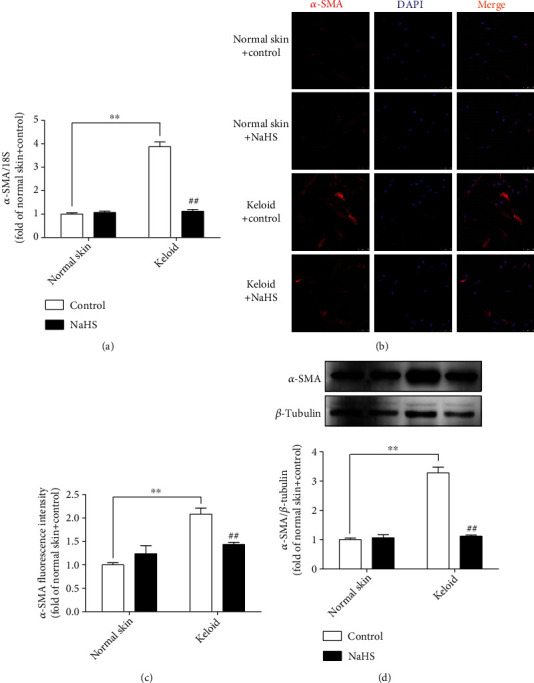
NaHS suppressed *α*-SMA expression in keloid fibroblasts. Skin fibroblasts were extracted from skin tissues of normal controls (normal skin) and scar tissue from patients with keloid (keloid), respectively. After pretreatment with NaHS (50 *μ*M) or the same amount of culture medium (control) for 4 h, the fresh culture medium was replaced. (a) After 12 h, *α*-SMA mRNA expression was detected with real-time PCR. (b) *α*-SMA was immunofluorescence stained with Cy3- (red) conjugated IgG. The nuclei were stained with DAPI (blue). Bar = 75 *μ*m. (c) Quantitative statistics of *α*-SMA fluorescence intensity. (d) *α*-SMA protein expression was detected by Western blot. ^∗∗^*P* < 0.01 vs. normal skin + control; ^##^*P* < 0.01 vs. keloid + control, *n* = 6.

*α*-SMA is one of the sensitive markers to indicate cells proliferation [26]. Therefore, the expression of *α*-SMA was detected in cells with real-time PCR, immunofluorescence, and Western blot. All these results consistently showed that there was more *α*-SMA expression in keloid fibroblasts than that in normal skin fibroblasts (*P* < 0.01), which was significantly suppressed by NaHS pretreatment (*P* < 0.01) (Figure 3). It demonstrated that exogenous H_2_S supplementation inhibited keloid fibroblast proliferation.

### 3.4. NaHS Inhibits PCNA Expression in Keloid Fibroblasts

**Figure 4:**
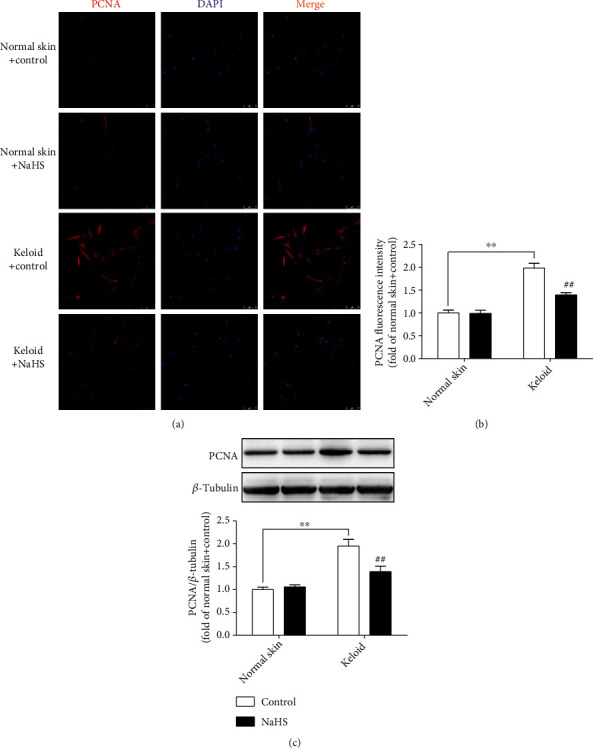
NaHS inhibited PCNA expression in keloid fibroblasts. Skin fibroblasts were extracted from skin tissues of normal controls (normal skin) and scar tissue from patients with keloid (keloid), respectively. After pretreatment with NaHS (50 *μ*M) or the same amount of culture medium (control) for 4 h, the fresh culture medium was replaced. (a) After 12 h, PCNA was immunofluorescence stained with Cy3- (red) conjugated IgG. The nuclei were stained with DAPI (blue). Bar = 75 *μ*m. (b) Quantitative statistics of PCNA fluorescence intensity. (c) Expression of PCNA protein was detected by Western Blot. ^∗∗^*P* < 0.01 vs. normal skin + control; ^##^*P* < 0.01 vs. keloid + control, *n* = 6.

PCNA, another sensitive indicator of skin fibroblast proliferation, plays an important role in DNA replication [27]. Therefore, PCNA was also detected to verify the influence of H_2_S on keloid fibroblast proliferation. Real-time PCR, immunofluorescence, and Western blot demonstrated that PCNA expression was higher in keloid fibroblasts than that in normal skin fibroblasts (*P* < 0.01), which was significantly suppressed by NaHS pretreatment (*P* < 0.01) (Figure 4), further indicating that exogenous H_2_S supplementation inhibited keloid fibroblast proliferation.

### 3.5. NaHS Blocks Collagen Synthesis in Keloid Fibroblasts

**Figure 5:**
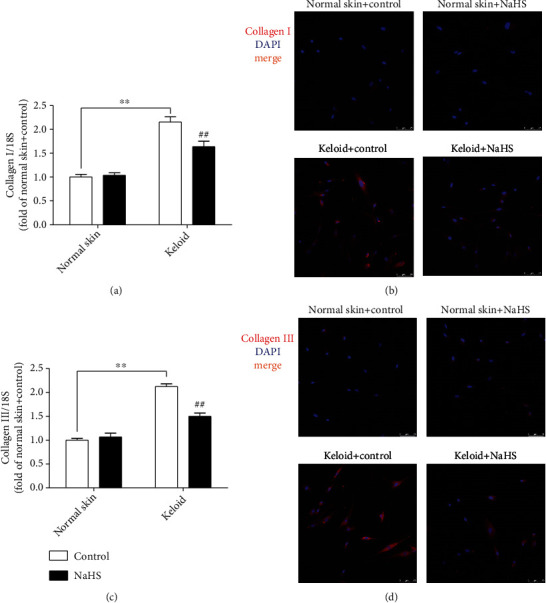
NaHS blocked collagen synthesis in keloid fibroblasts. Skin fibroblasts were extracted from skin tissues of normal controls (normal skin) and scar tissue from patients with keloid (keloid), respectively. After pretreatment with NaHS (50 *μ*M) or the same amount of culture medium (control) for 4 h, the fresh culture medium was replaced. (a) After 12 h, collagen I mRNA expression was detected with real-time PCR. (b) Collagen I was immunofluorescence stained with Cy3- (red) conjugated IgG. The nuclei were stained with DAPI (blue). Bar = 75 *μ*m. (c) Collagen III mRNA expression was detected with real-time PCR. (d) Collagen III was immunofluorescence stained with Cy3- (red) conjugated IgG. The nuclei were stained with DAPI (blue). Bar = 75 *μ*m. ^∗∗^*P* < 0.01 vs. normal skin + control; ^##^*P* < 0.01 vs. keloid + control, *n* = 6.

Then, the content of collagen I and collagen III was measured to evaluate the influence of H_2_S on collagen synthesis. Both real-time PCR and immunofluorescence verified that compared with normal skin fibroblasts, the expression of collagen I and collagen III in keloid fibroblasts was significantly higher than that in normal skin fibroblasts (*P* < 0.01), which was reduced by NaHS pretreatment (*P* < 0.01) (Figure 5). It suggested that exogenous H_2_S supplementation blocked collagen synthesis in keloid fibroblasts.

### 3.6. NaHS Attenuates Oxidative Stress in Keloid Fibroblasts

**Figure 6:**
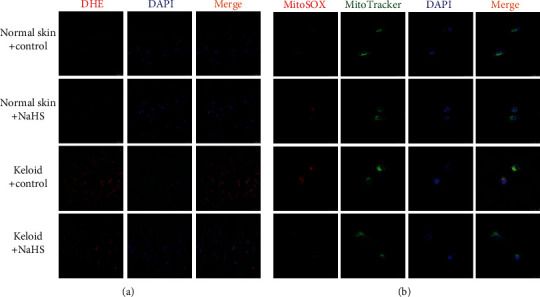
NaHS attenuated oxidative stress in keloid fibroblasts. Skin fibroblasts were extracted from skin tissues of normal controls (normal skin) and scar tissue from patients with keloid (keloid), respectively. After pretreatment with NaHS (50 *μ*M) or the same amount of culture medium (control) for 4 h, the fresh culture medium was replaced. (a) After 12 h, intracellular superoxide anion was stained by DHE (red). The nuclei were stained with DAPI (blue). Bar = 50 *μ*m. (b) Mitochondrial superoxide was stained with MitoSOX (red). The mitochondria were stained with MitoTracker (green). The nuclei were stained with DAPI (blue). Bar = 25 *μ*m.

DHE staining and MitoSOX staining were applied to detect the level of intracellular superoxide anion and mitochondrial superoxide, respectively. Compared with normal skin fibroblasts, red fluorescence by DHE and MitoSOX staining in keloid fibroblasts was stronger, which was weakened by NaHS pretreatment (Figure 6), demonstrating that intracellular superoxide anion and mitochondrial superoxide were both inhibited by NaHS. It suggested that exogenous H_2_S supplementation attenuated oxidative stress in keloid fibroblasts.

### 3.7. NaHS Improves Mitochondrial Membrane Potential in Keloid Fibroblasts

**Figure 7:**
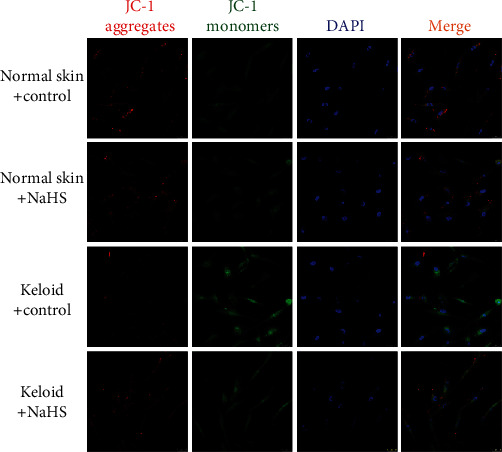
NaHS improved mitochondrial membrane potential in keloid fibroblasts. Skin fibroblasts were extracted from skin tissues of normal controls (normal skin) and scar tissue from patients with keloid (keloid), respectively. After pretreatment with NaHS (50 *μ*M) or the same amount of culture medium (control) for 4 h, the fresh culture medium was replaced. After 12 h, mitochondrial membrane potential was detected by JC-1 staining. JC-1 aggregates (red) and JC-1 monomers (green) were observed. The nuclei were stained with DAPI (blue). Bar = 25 *μ*m.

Decreased mitochondrial membrane potential (Δ*ψ*m) might contribute to mitochondrial injury and ROS accumulation [28]. Red fluorescence by JC-1 staining indicated normal mitochondria with higher Δ*ψ*m, while green indicated impaired mitochondria with lower Δ*ψ*m. Our experiment demonstrated that compared with normal skin fibroblasts, green fluorescence was increased, but red fluorescence was decreased in keloid fibroblasts, while NaHS pretreatment significantly enhanced red and weakened green fluorescence (Figure 7), suggesting that exogenous H_2_S supplementation improved mitochondrial membrane potential to alleviate mitochondrial damage in keloid fibroblasts.

### 3.8. NaHS Alleviates Necroptosis in Keloid Fibroblasts

**Figure 8:**
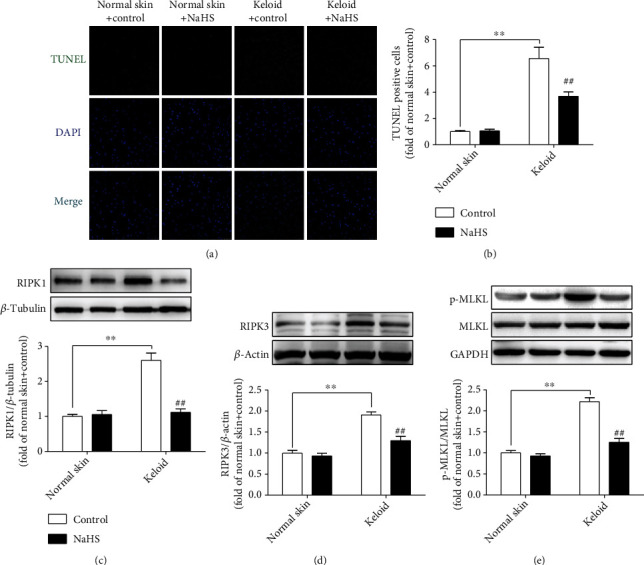
NaHS alleviated necroptosis in keloid fibroblasts. Skin fibroblasts were extracted from skin tissues of normal controls (normal skin) and scar tissue from patients with keloid (keloid), respectively. After pretreatment with NaHS (50 *μ*M) or the same amount of culture medium (control) for 4 h, the fresh culture medium was replaced. (a) After 12 h, necroptotic cells were marked with TUNEL staining (green). The nuclei were stained with DAPI (blue). Bar = 50 *μ*m. (b) Quantitative statistics of TUNEL-positive cells. (c–e) Expression of RIPK1, RIPK3, and MLKL was detected by Western blot. ^∗∗^*P* < 0.01 vs. normal skin + control; ^##^*P* < 0.01 vs. keloid + control, *n* = 6.

Due to the intercellular genomic DNA breakage and 3′ OH exposing at the early stage, apoptotic cells were able to be marked by TUNEL staining, which is also one characteristic of necroptosis [29]. Necroptosis is strictly regulated by multiple molecules. RIPK1 and RIPK3, two key signal molecules to mediate necroptosis, are commonly considered specific indicators of necroptosis. In addition, MLKL is a core substrate of RIPK3 downstream to mediate necroptosis. Therefore, TUNEL staining and the above proteins measurement were applied to evaluate cell necroptosis. Compared with normal skin fibroblasts, there were more TUNEL staining-positive cells, higher RIPK1 and RIPK3 expressions as well as MLKL phosphorylation in keloid fibroblasts (*P* < 0.01), which were all reversed by NaHS pretreatment (*P* < 0.01) (Figure 8). These data indicated that exogenous H_2_S supplementation alleviated necroptosis in keloid fibroblasts.

### 3.9. NaHS Inhibits TGF-*β*_1_-Stimulated Skin Fibroblast Migration after Scratch

**Figure 9:**
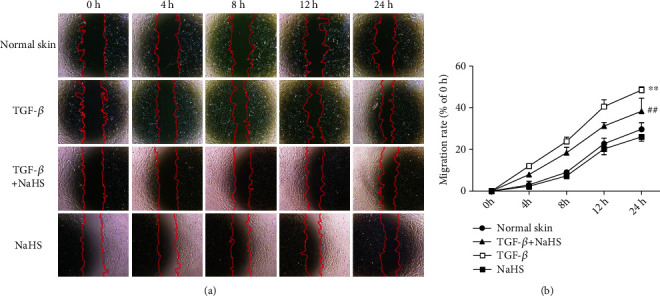
NaHS inhibited TGF-*β*_1_-stimulated skin fibroblast migration after scratch. Skin fibroblasts were extracted from skin tissues of normal controls (normal skin). After pretreatment with NaHS (50 *μ*M) for 4 h, skin fibroblasts were stimulated with TGF-*β*_1_ (10 ng/mL) for 12 h. The cells were scratched with 10 *μ*L pipette tip. (a) Photos were taken after scratch for 0 h, 4 h, 8 h, 12 h, and 24 h. (b) The percentage of cell migration area to initial scratch area at different time. ^∗∗^*P* < 0.01 vs. normal skin; ^##^*P* < 0.01 vs. TGF-*β*_1_, *n* = 6.

The above results demonstrated that exogenous H_2_S supplementation inhibited keloid fibroblast proliferation. However, whether the above effects could be extended to other dermatofibrotic diseases is unknown. Therefore, normal skin fibroblasts were extracted and stimulated with TGF-*β*_1_. Skin fibroblast migration with TGF-*β*_1_ stimulation after scratch was faster than normal skin fibroblasts (*P* < 0.01), which was reversed by NaHS pretreatment (*P* < 0.01) (Figure 9). It suggested that exogenous H_2_S supplementation inhibited TGF-*β*_1_ stimulated skin fibroblast migration after scratch.

### 3.10. NaHS Suppresses *α*-SMA Expression in TGF-*β*_1_-Stimulated Skin Fibroblasts

**Figure 10:**
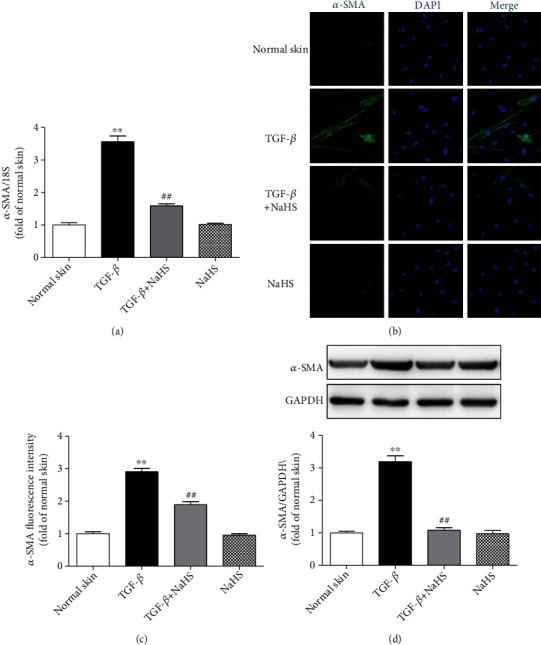
NaHS suppressed *α*-SMA expression in TGF-*β*_1_-stimulated fibroblasts. Skin fibroblasts were extracted from skin tissues of normal controls (normal skin). After pretreatment with NaHS (50 *μ*M) for 4 h, skin fibroblasts were stimulated with TGF-*β*_1_ (10 ng/mL) for 12 h. (a) *α*-SMA mRNA expression was detected with real-time PCR. (b) *α*-SMA was immunofluorescence stained with Alexa Fluor 488- (green) conjugated IgG. The nuclei were stained with DAPI (blue). Bar = 25 *μ*m. (c) Quantitative statistics of *α*-SMA fluorescence intensity. (d) *α*-SMA protein expression was detected by Western blot. ^∗∗^*P* < 0.01 vs. normal skin; ^##^*P* < 0.01 vs. TGF-*β*_1_, *n* = 6.

Compared with normal skin fibroblasts, there were more *α*-SMA expression after TGF-*β*_1_ stimulation (*P* < 0.01), which was decreased by NaHS pretreatment (*P* < 0.01) (Figure 10), suggesting that exogenous H_2_S supplementation suppressed TGF-*β*_1_-stimulated skin fibroblast proliferation.

### 3.11. NaHS Inhibits PCNA Expression in TGF-*β*_1_-Stimulated Skin Fibroblasts

**Figure 11:**
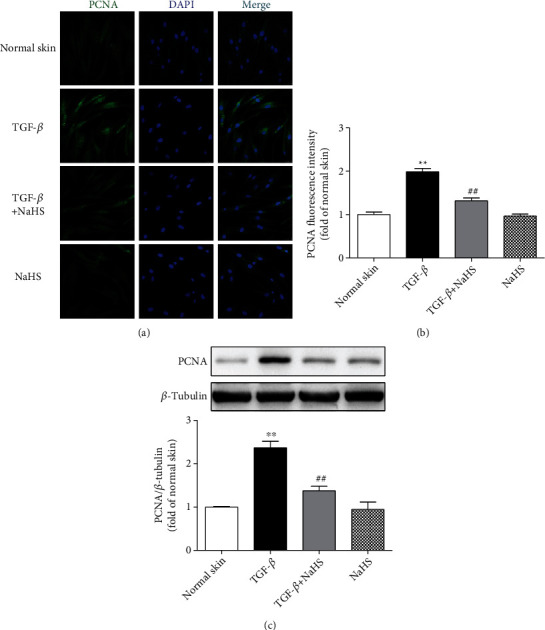
NaHS inhibited PCNA expression in TGF-*β*_1_-stimulated fibroblasts. Skin fibroblasts were extracted from skin tissues of normal controls (normal skin). After pretreatment with NaHS (50 *μ*M) for 4 h, skin fibroblasts were stimulated with TGF-*β*_1_ (10 ng/mL) for 12 h. (a) PCNA was immunofluorescence stained with Alexa Fluor 488- (green) conjugated IgG. The nuclei were stained with DAPI (blue). Bar = 25 *μ*m. (b) Quantitative statistics of PCNA fluorescence intensity. (c) PCNA protein expression was detected by Western blot. ^∗∗^*P* < 0.01 vs. normal skin; ^##^*P* < 0.01 vs. TGF-*β*_1_, *n* = 6.

Compared with normal skin fibroblasts, there were higher PCNA expression after TGF-*β*_1_ stimulation (*P* < 0.01), which was inhibited by NaHS pretreatment (*P* < 0.01) (Figure 11), further indicating that exogenous H_2_S supplementation suppressed TGF-*β*_1_-stimulated skin fibroblast proliferation.

### 3.12. NaHS Blocks Collagen Synthesis in TGF-*β*_1_-Stimulated Skin Fibroblasts

**Figure 12:**
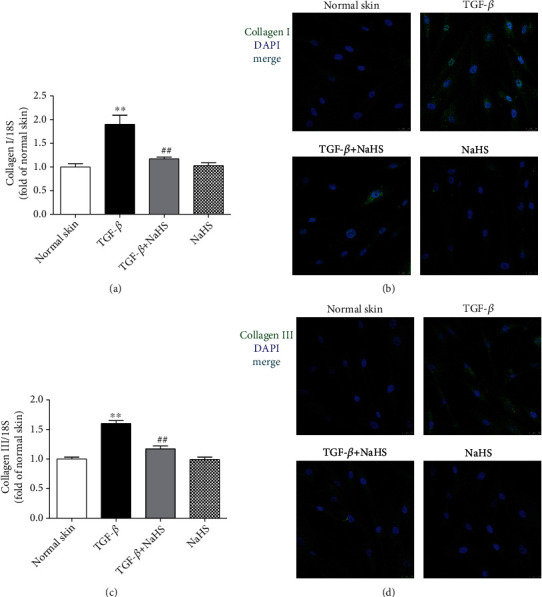
NaHS prevented collagen synthesis in TGF-*β*_1_-stimulated fibroblasts. Skin fibroblasts were extracted from skin tissues of normal controls (normal skin). After pretreatment with NaHS (50 *μ*M) for 4 h, skin fibroblasts were stimulated with TGF-*β*_1_ (10 ng/mL) for 12 h. (a) Collagen I mRNA expression was detected with real-time PCR. (b) Collagen I was immunofluorescence stained with Alexa Fluor 488- (green) conjugated IgG. The nuclei were stained with DAPI (blue). Bar = 25 *μ*m. (c) Collagen III mRNA expression was detected with real-time PCR. (d) Collagen III was immunofluorescence stained with Alexa Fluor 488- (green) conjugated IgG. The nuclei were stained with DAPI (blue). Bar = 25 *μ*m. ^∗∗^*P* < 0.01 vs. normal skin; ^##^*P* < 0.01 vs. TGF-*β*_1_, *n* = 6.

Compared with normal skin fibroblasts, there were more collagen I and collagen III expressions after TGF-*β*_1_ stimulation (*P* < 0.01), which was suppressed by NaHS pretreatment (*P* < 0.01) (Figure 12), indicating that exogenous H_2_S supplementation blocked TGF-*β*_1_-stimulated skin fibroblast collagen synthesis.

### 3.13. NaHS Attenuates Oxidative Stress in TGF-*β*_1_-Stimulated Skin Fibroblasts

**Figure 13:**
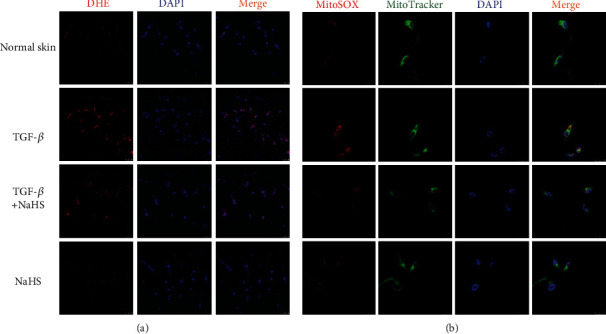
NaHS attenuated oxidative stress in TGF-*β*_1_-stimulated skin fibroblasts. Skin fibroblasts were extracted from skin tissues of normal controls (normal skin). After pretreatment with NaHS (50 *μ*M) for 4 h, skin fibroblasts were stimulated with TGF-*β*_1_ (10 ng/mL) for 12 h. (a) The intracellular superoxide anion was stained by DHE (red). The nuclei were stained with DAPI (blue). Bar = 50 *μ*m. (b) Mitochondrial superoxide was stained with MitoSOX (red). The mitochondria were stained with MitoTracker (green). The nuclei were stained with DAPI (blue). Bar = 25 *μ*m.

Compared with normal skin fibroblasts, TGF-*β*_1_ stimulation enhanced red fluorescence in skin fibroblasts, which was weakened by NaHS pretreatment (Figure 13), and demonstrated that intracellular superoxide anion and mitochondrial superoxide were inhibited by NaHS. It suggested that exogenous H_2_S supplementation attenuated oxidative stress in TGF-*β*_1_-stimulated skin fibroblasts.

### 3.14. NaHS Alleviates Necroptosis in TGF-*β*_1_-Stimulated Skin Fibroblasts

**Figure 14:**
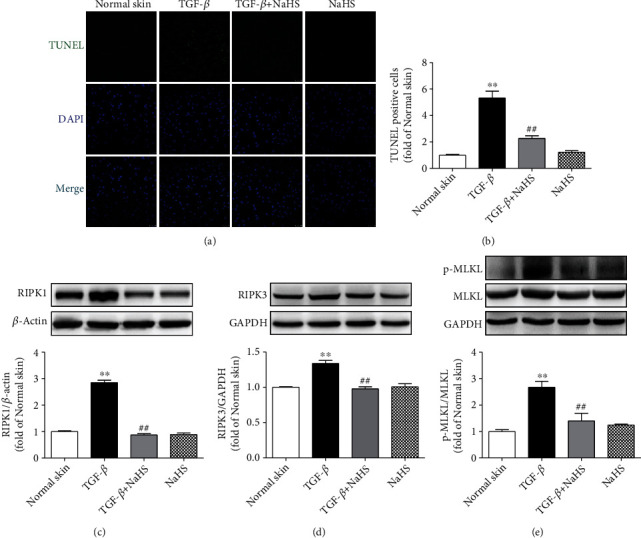
NaHS alleviated necroptosis in TGF-*β*_1_-stimulated skin fibroblasts. Skin fibroblasts were extracted from skin tissues of normal controls (normal skin). After pretreatment with NaHS (50 *μ*M) for 4 h, skin fibroblasts were stimulated with TGF-*β*_1_ (10 ng/mL) for 12 h. (a) Necroptotic cells were marked with TUNEL staining (green). The nuclei were stained with DAPI (blue). Bar = 50 *μ*m. (b) Quantitative statistics of TUNEL-positive cells. (c–e) Expression of RIPK1, RIPK3, and MLKL was detected by Western blot. ^∗∗^*P* < 0.01 vs. normal skin; ^##^*P* < 0.01 vs. TGF-*β*_1_, *n* = 6.

Compared with normal skin fibroblasts, TGF-*β*_1_ stimulation increased the positive rate of TUNEL staining and upregulated RIPK1 and RIPK3 expressions as well as MLKL phosphorylation (*P* < 0.01), which were all reversed by NaHS pretreatment (*P* < 0.01) (Figure 14). These results suggested that exogenous H_2_S supplementation alleviated necroptosis in TGF-*β*_1_-stimulated skin fibroblasts.

## 4. Discussion

As an endogenous gasotransmitter, H_2_S plays an important role in a variety of pathophysiological processes. H_2_S dysfunction is reported to be involved in the pathogenesis of hypertension, diabetes, atherosclerosis, and tumor [30–32]. Previous studies showed that excessive H_2_S production induced thyroid malignancy, deteriorated pancreatitis, and lung injury [33, 34], while impaired H_2_S production promoted obesity and aggravated insulin resistance [35]. In addition, H_2_S content was significantly decreased in patients with psoriasis, which was negatively correlated with the severity of clinical symptoms [36, 37]. H_2_S also facilitated melanoma cell apoptosis and inhibited tumor growth by upregulating Fas-associated protein with a novel death domain (FADD) [38]. Exogenous H_2_S suppressed melanoma cells migration and invasion to delay melanoma progression and metastasis formation [39]. Interestingly, a novel drug carrier nanofibrous membrane, blending the recombinant spider silk protein and NaHS by electrospun, promoted skin wound healing [40]. These studies suggest that H_2_S has a potential effect on skin diseases.

Keloid is a skin benign fibroproliferative disease, and the definite pathogenesis of keloid is not clear. We found that the H_2_S level in the plasma and skin of patients with keloid was notably decreased, suggesting that H_2_S production impairment might be involved in human skin fibroblast proliferation. Previous studies have verified that H_2_S improved multiple fibrotic diseases. Specifically, exogenous H_2_S prevented myocardial fibrosis during myocardial infarction, hypertension, and diabetes [41]. H_2_S also attenuated vascular fibrosis by regulating endothelial mesenchymal transition (EndMT) [42]. H_2_S alleviated hypoxia-induced chronic renal fibrosis *via* restoring ten-eleven translocations (TET), reversing DNA methylation, and enhancing Klotho gene expression [43]. Our present study found that NaHS pretreatment slowed down keloid fibroblast migration, decreased the expression of *α*-SMA, PCNA, collagen I, and collagen III. It suggested that exogenous H_2_S supplementation inhibited keloid fibroblast proliferation. Then we further explored the possible effect of H_2_S on some other fibrotic diseases. It is widely known that the excess activation of TGF-*β*_1_ signaling pathway contributed to the occurrence and development of dermatofibrotic diseases including keloid [44]. Therefore, TGF-*β*_1_ stimulation is a common experimental strategy to induce skin fibroblast proliferation and imitate dermatofibrotic diseases. Our results showed that TGF-*β*_1_ stimulation accelerated skin fibroblast migration and upregulated the expression of *α*-SMA, PCNA, collagen I, and collagen III, suggesting that TGF-*β*_1_ successfully induced critical characteristics of skin fibrosis *in vitro*. Similar to the preventive effects against keloid fibroblasts, NaHS pretreatment also inhibited cell migration and suppressed the expression of *α*-SMA, PCNA, collagen I, and collagen III in TGF-*β*_1_-stimulated skin fibroblasts. Taken together, the above evidence indicates that H_2_S possibly has a powerful therapeutic effect on skin fibrosis, which is conducive to developing novel ideas for the prevention and treatment of dermatofibrotic diseases.

Previous studies verified that H_2_S attenuated oxidative stress, regulated glucose and fat metabolism, and improved mitochondrial function to alleviate liver cirrhosis [45]. H_2_S inhibited inflammation and oxidative stress, reduced the expression of Toll like receptor 4 (TLR4), NOD like receptor family pyrin domain containing 3 (NLRP3), and caspase-1, improved the renal function and renal histopathological changes, and alleviated kidney injury in sepsis-associated acute kidney injury (SA-AKI) [46]. H_2_S also inhibited oxidative stress to correct the dysfunction in neurodegenerative diseases [47]. H_2_S attenuated paraquat-induced acute liver injury *via* enhancing antioxidant capacity, regulating mitochondrial function, and inhibiting NLRP3 inflammasome activation [48]. All these studies indicated that the antioxidant effect of H_2_S might be one of the common mechanisms to improve fibrosis in various organs and tissues. DHE, MitoSOX, and JC-1 staining in our experiment confirmed that NaHS pretreatment decreased intracellular superoxide anion and mitochondrial superoxide but increased mitochondrial membrane potential in keloid fibroblasts and TGF-*β*_1_-stimulated normal skin fibroblasts, suggesting that exogenous H_2_S supplementation attenuated oxidative stress. These results indicated that the antiproliferation effect of H_2_S on skin fibroblasts might be ascribed to oxidative stress inhibition.

Intracellular ROS accumulation can induce cell necroptosis, which is a new type of programmed cell death with the characteristics of both apoptosis and necrosis. Necroptosis plays an important role in maintaining cell homeostasis, regulating tumor immunity and inflammatory response, and mediating ischemic injury and several other pathophysiological processes [49, 50]. As two important mediators of necroptosis, RIPK1 combines with RIPK3 to form necrosomes and phosphorylate MLKL, which then transfers to cell membrane and ruptures it, and exaggerates cell necroptosis finally [51]. Due to the enhanced oxidative stress, necroptosis was consequently increased in mice with chronic hepatitis [52]. Some researchers found that ROS accumulation in melanocytes' mitochondria promoted necrosome formation and aggravated necroptosis in the pathogenesis of vitiligo [53]. Our present experiment showed that NaHS pretreatment downregulated the expression of RIPK1 and RIPK3, inhibited MLKL phosphorylation, and decreased the TUNEL staining-positive rate in keloid fibroblasts and TGF-*β*_1_-stimulated normal skin fibroblasts, suggesting that exogenous H_2_S supplementation alleviated necroptosis in both keloid fibroblasts and TGF-*β*_1_-stimulated normal skin fibroblasts. However, besides expression of RIPK1, RIPK3, and MLKL, some other proteins including cleaved caspase 3, necrosomes, and inflammasomes had not been detected in our present study. Actually, more necroptosis proteins will be a great benefit to validate the effect of H_2_S on necroptosis in skin fibroblasts. This is one limitation of our present study. Considering the powerful antioxidant effects, the suppressive ability of necroptosis by H_2_S may be attributed to the inhibition of oxidative stress. Interestingly, inhibition of skin fibroblasts necroptosis by H_2_S seems to contradict with the preventive effects on proliferation. Actually, a large amount of cellular contents will be released after necroptosis, such as inflammatory factors, cell metabolites, and other substances, which all further promote cell proliferation. Recently, some researchers found that H_2_S attenuated necroptosis depending on sirtuin3 enhancement to suppress excessive proliferation in hypoxia-induced cardiac fibroblasts [25]. That is to say that the mutual regulation between cell death and proliferation is very complex. Therefore, the inhibitory mechanism of H_2_S on skin fibroblast proliferation may involve some other reactions, such as extracellular matrix degradation, inflammation aggravation, and cytokine release, which need future research.

In conclusion, H_2_S production was impaired in the plasma and skin of patients with keloid. Exogenous H_2_S supplementation suppressed proliferation in keloid fibroblasts and TGF-*β*_1_-stimulated normal skin fibroblasts. The suppressive mechanisms may be related to oxidative stress alleviation and necroptosis inhibition. Our present study is of benefit to provide a novel strategy for the prevention and treatment of keloid and some other dermatofibrotic diseases.

## Data Availability

The data used to support the finding of this study are available from the corresponding authors upon request.
